# Incident acute pseudogout and prior bisphosphonate use: Matched case–control study in the UK-Clinical Practice Research Datalink

**DOI:** 10.1097/MD.0000000000006177

**Published:** 2017-03-24

**Authors:** Edward Roddy, Sara Muller, Zoe Paskins, Samantha L. Hider, Milisa Blagojevic-Bucknall, Christian D. Mallen

**Affiliations:** Research Institute for Primary Care and Health Sciences, Keele University, Keele, Staffordshire, UK.

**Keywords:** acute pseudogout, bisphosphonates, calcium pyrophosphate crystal deposition, case–control study, primary care

## Abstract

Oral bisphosphonates are the most commonly used drugs to treat postmenopausal osteoporosis. Acute pseudogout is anecdotally reported to occur following bisphosphonate initiation but empirical data are lacking. We investigated whether treatment with oral bisphosphonates is a risk factor for incident acute pseudogout.

A matched case–control study was undertaken using data from the UK-Clinical Practice Research Datalink. Adults who consulted for incident acute pseudogout between 1987 and 2012 were each matched for gender, age at pseudogout diagnosis, and general practice to up to 4 control subjects without pseudogout. The exposure of interest was a prescription for an oral bisphosphonate issued within the 60-day period prior to the date of incident acute pseudogout. Associations between incident acute pseudogout and prior bisphosphonate prescription were examined using conditional logistic regression, adjusting for hyperparathyroidism, osteoarthritis, rheumatoid arthritis, hemochromatosis, hypophosphatasia, and prescriptions for diuretics and oral corticosteroids.

Two thousand eleven acute pseudogout cases were compared with 8013 matched controls without acute pseudogout (mean age [standard deviation] 72 years [14]; 52% male). One hundred twenty-three cases (6.1%) had received an oral bisphosphonate prescription in the 60-day exposure period compared with 305 controls (3.8%) (adjusted incidence rate ratio [IRR] 1.33; 95% confidence interval [CI] 1.05–1.69). This association was stronger in females (adjusted IRR 1.49; 95% CI 1.15–1.94) and was nonsignificant in males (0.83; 0.48–1.44).

Incident acute pseudogout was associated with prescription of an oral bisphosphonate in the preceding 60 days. Prescribers should be aware of acute pseudogout as a possible side effect of bisphosphonate treatment. Further research is needed to explore the risks conferred by different bisphosphonates and the mechanism underlying this association.

## 1 Introduction

Calcium pyrophosphate crystal deposition (CPPD) is a common idiopathic age-related phenomenon. Clinical presentations are varied and include asymptomatic radiographic chondrocalcinosis, chronic arthropathy, and, most dramatically, acute attacks of joint pain and swelling which most commonly affect the knee (acute pseudogout/acute calcium pyrophosphate [CPP] crystal arthritis).^[1]^ The prevalence of radiographic chondrocalcinosis in adults aged over 40 years is 4.5%, and rises sharply with age, peaking at 18% in people aged over 80 years.^[2]^ Less commonly, CPPD can occur secondary to metabolic disorders, namely hemochromatosis, hyperparathyroidism, hypomagnesemia and hypophosphatasia, or rarer familial aggregation.^[3–7]^ Diuretic use has been suggested to be a risk factor for radiographic chondrocalcinosis and acute pseudogout, and is thought to act via renal magnesium loss.^[2,8]^

Several case reports describe the occurrence of acute pseudogout following initiation of treatment with oral and intravenous bisphosphonates,^[9–14]^ raising the possibility that bisphosphonate therapy is also a risk factor for CPPD. Bisphosphonates are synthetic analogs of pyrophosphate and are potent inhibitors of bone resorption, achieved by the inhibition of osteoclasts.^[15]^ They are most commonly used as treatment for postmenopausal osteoporosis but other licensed indications include other forms of osteoporosis, Paget's disease, hypercalcemia, and metastatic bone disease.^[16,17]^ Oral alendronic acid, the most commonly prescribed bisphosphonate, accounted for 7.4 million items dispensed in the community in England in 2014.^[18]^ The most commonly experienced adverse events are upper gastrointestinal symptoms although rarer side effects include osteonecrosis of the jaw and atypical femoral fractures.^[19,20]^ Several plausible biological mechanisms exist via which bisphosphonates could predispose to formation and shedding of CPP crystals. Bisphosphonates can lower serum calcium levels acutely, a phenomenon described in 3 published case reports of bisphosphonate-induced acute pseudogout,^[10,12,13]^ which could theoretically precipitate the shedding of CPP crystals from articular cartilage into synovial fluid.^[21]^ Disodium etidronate inhibits renal phosphate excretion, raising serum phosphate levels and thereby predisposing to CPPD.^[22]^ Furthermore, bisphosphonates are pyrophosphate analogs and impair CPP crystal dissolution via an inhibitory effect on the pyrophosphatase activity of alkaline phosphatase.^[23]^

Despite these potential mechanisms, there have been no empirical research studies to examine the occurrence of acute pseudogout as a complication of bisphosphonate treatment. The aim of this matched case–control study was to investigate whether treatment with oral bisphosphonates is a risk factor for incident acute pseudogout in a primary care population, and to examine whether different oral bisphosphonates confer the same risk.

## 2 Methods

### 2.1 CPRD

The UK Clinical Practice Research Datalink (CPRD) is the largest database of electronic primary care health records in the world. It is a repository of clinical data from over 600 general practices in the England and Wales, covering 5.5 million people, and includes records of consultations, prescriptions, referrals, and investigations. To be included in CPRD, the quality of data provided by contributing practices must meet stringent criteria and be considered to be “up to standard” for research purposes.^[24]^ Previous studies of the epidemiology of inflammatory conditions including crystal arthropathies have been undertaken using data from CPRD^[25–30]^ and a high validity of diagnosis has been reported.^[24,31]^ The study was approved by the CPRD Independent Scientific Advisory Committee (protocol 13_103).

### 2.2 Sample selection

#### 2.2.1 Cases

The outcome of interest was incident acute pseudogout. Cases were selected as all adults aged 18 years and over with a first recorded Read-coded diagnosis of pseudogout between March 1, 1987 and December 31, 2012. In those with multiple codes for pseudogout, only the first occurrence of the condition was included in analyses. Pseudogout was defined by the single Read code N02 14.^[8]^

#### 2.2.2 Controls

Each case was individually matched to up to 4 individuals who did not have a diagnosis of pseudogout up to the “index date” of their matched case (i.e., the date on which the case was diagnosed with pseudogout) using concurrent sampling. Matching was on the basis of general practice, gender, and age (within 3 years) at the index date. Controls were also required to have consulted with the practice in the year that the matched case was diagnosed with pseudogout, thus ensuring that controls were active members of the practice and reducing the possibility of artificially inflating the consultation rates of cases by comparing them to “nonconsulters.”

In order to ensure high-quality data in the exposure period, all cases and controls were required to have 6 months of up-to-standard data prior to the index date.

### 2.3 Exposure

The exposure of interest was a prescription for an oral bisphosphonate issued within the 60-day period prior to the index date. Codes used to define oral bisphosphonates are available from the authors on request. Intravenous bisphosphonates were not considered as these are not prescribed in primary care in the UK^[18]^ and, as a result, are not routinely recorded in CPRD.

### 2.4 Potential confounding variables

Potential confounders of the association between oral bisphosphonates and pseudogout were considered to be a diagnosis of hyperparathyroidism, osteoarthritis (OA), rheumatoid arthritis (RA), hemochromatosis or hypophosphatasia, or a prescription for diuretics (loop or thiazide) or oral corticosteroids. Medical diagnoses were identified by Read code. Since these medical conditions are chronic in nature, entry of a Read code for these conditions recorded at any point prior to the index date was considered to indicate presence of the disease. Prescription records were searched for prescription of diuretics or oral corticosteroids in the 60 days prior to the index date. Read codes and prescription codes used to identify medical diagnoses and medications are available from the authors on request.

### 2.5 Power calculation

An a priori feasibility count undertaken by the CPRD knowledge center estimated that 2147 eligible cases of pseudogout would be identified in the study period. Matching these cases to controls in a ratio of 1:4, and assuming a rate of exposure to bisphosphonates in the controls of 1.6%, along with a correlation of exposure between cases and controls of 0.2% and 5% significance level, it was estimated that the study would have approximately 98% power to detect an odds ratio of 2.0.

### 2.6 Statistical analysis

Unadjusted conditional logistic regression was used to assess the association between oral bisphosphonate prescriptions and incident acute pseudogout status in all analyses. Analyses were first conducted for all oral bisphosphonates combined and then separately for individual oral bisphosphonate preparations (alendronic acid, disodium etidronate, ibandronic acid, risedronate sodium, sodium clodronate, and tiludronate disodium). Models were then adjusted for hyperparathyroidism, OA, RA, hemochromatosis, hypophosphatasia, and prescriptions in the 60 days prior to the index date of diuretics and oral corticosteroids. As control subjects were identified by risk set sampling, the odds ratios from all analyses can be interpreted, and so are presented, as incidence rate ratios (IRRs) with 95% confidence intervals (95% CIs).

Three sensitivity analyses were performed. First, the primary analysis of the association between prescription of all oral bisphosphonates in the 60 days prior to the index date and incident acute pseudogout was repeated after excluding matched case and controls in whom the pseudogout case had received their first bisphosphonate prescription >60 days prior to the index date in order to investigate whether any association arises from cumulative or incident bisphosphonate exposure. Second, in order to address the possibility of misclassification bias (i.e., patients with gout being misclassified as having pseudogout), the primary analyses were repeated having excluded any individuals (cases or controls) who had a diagnosis of gout prior to the index date. Third, in order to investigate who may be susceptible to any increased risk of incident pseudogout following prescription of bisphosphonates, models were fitted with interaction terms separately for age and gender where any significant associations were found in the main analyses. The significance of interaction terms was assessed using likelihood ratio tests.

All analyses were conducted in Stata MP2 14.1.^[32]^

## 3 Results

During the study period, 2011 individuals with an incident diagnosis of pseudogout were identified and successfully matched for gender, age at pseudogout diagnosis, and general practice to 8013 control subjects without pseudogout (Table 1). Mean age of pseudogout cases was 72.0 years (standard deviation 13.5) and 1051 (52.3%) were male. Hyperparathyroidism, OA, RA, prescription of oral corticosteroids, and gout were more prevalent in the pseudogout cases than control subjects (Table 1).

**Table 1:**
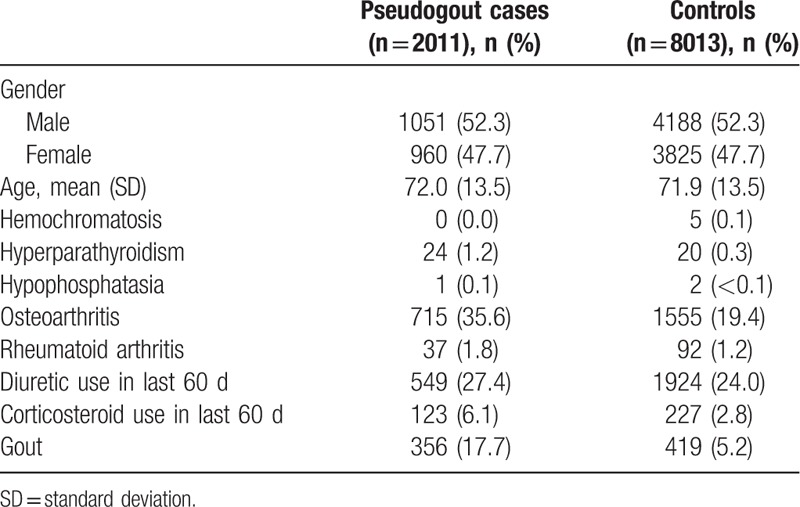
Participant characteristics.

One hundred twenty-three pseudogout cases (6.1%) had received a prescription for an oral bisphosphonate in the 60-day period preceding the index date compared with 305 (3.8%) controls (unadjusted IRR 1.69; 95% CI 1.35–2.12). After adjustment for hyperparathyroidism, OA, RA, and prescriptions for diuretics and oral corticosteroids, this association attenuated slightly (IRR 1.33; 95% CI 1.05–1.69). Owing to the very small numbers of patients identified with hemochromatosis and hypophosphatasia (Table 1), these diagnoses were not included as covariates in the model. In undertaking the first sensitivity analysis to investigate the effect of cumulative or incident bisphosphonate exposure, we found that all pseudogout cases initiated a bisphosphonate within the period 60 days prior to the index date, therefore replicating the main analysis.

The most commonly used oral bisphosphonate was alendronic acid (Table 2). Eighty-four pseudogout cases (4.2%) received a prescription for alendronic acid in the 60-day period preceding the index date compared with 211 (2.6%) controls (adjusted IRR 1.36; 95% CI 1.03–1.79). Positive associations between prescription during the 60 days prior to the index date and incident pseudogout were seen for disodium etidronate, risedronate sodium, and sodium clodronate whereas a negative association was seen for ibandronic acid. These associations were not statistically significant although the numbers of prescriptions for these drugs were small. No participants were prescribed tiludronate disodium.

**Table 2:**
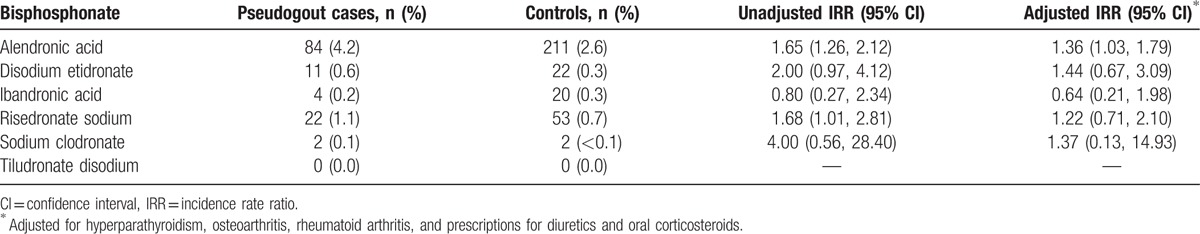
Association between incident pseudogout and prescription of individual oral bisphosphonates within the preceding 60 days.

After excluding participants with a diagnosis of gout prior to the index date, the significant associations with any oral bisphosphonate (adjusted IRR 1.43; 95% CI 1.11–1.85) and alendronic acid (1.53; 1.15–2.06) in the 60 days prior to pseudogout were slightly stronger (Table 3).

**Table 3:**
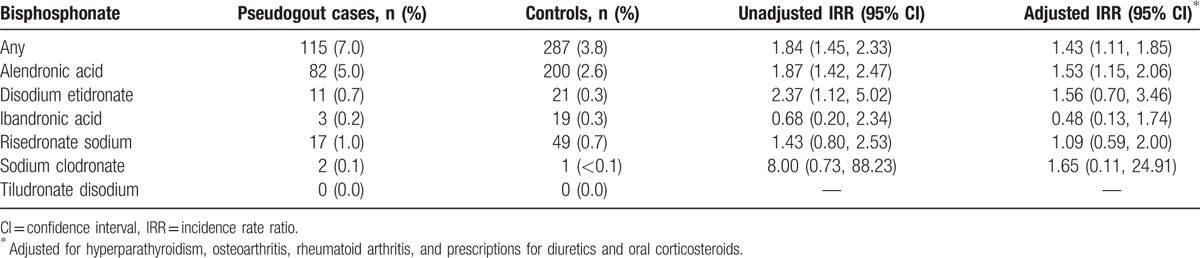
Sensitivity analysis excluding cases and controls with a diagnosis of gout prior to the index date.

The association between prescription of any oral bisphosphonate in the 60 days prior to index date was stronger in females (adjusted IRR 1.49; 95% CI 1.15–1.94) and was nonsignificant in males (0.83; 0.48–1.44) (*P* value for interaction; unadjusted 0.121; adjusted 0.048) and diminished with age, although there was no significant association in any age-group (*P* value for interaction; unadjusted 0.610, adjusted 0.781) (Table 4). Similar associations were seen for prescription of alendronic acid in the 60 days prior to the index date (data not shown).

**Table 4:**
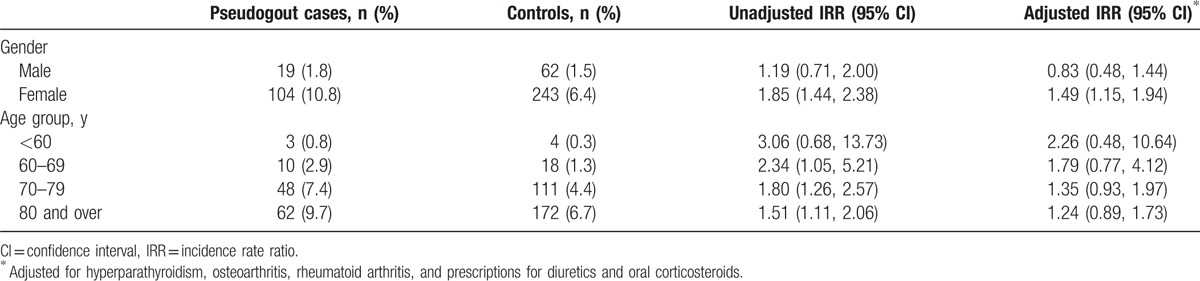
Association between incident pseudogout and prescription of any oral bisphosphonate within the preceding 60 days by age and gender.

## 4 Discussion

This is the first empirical epidemiological study to determine an association between bisphosphonates and incident acute pseudogout. We found that people who developed incident acute pseudogout were more likely to have received a prescription for an oral bisphosphonate in the preceding 60 days than control subjects without pseudogout, matched for age, gender, and general practice. This association persisted after adjustment for confounding comorbid conditions and medications. Similar associations were seen between incident pseudogout and prior prescription of alendronic acid, disodium etidronate, risedronate sodium, and sodium clodronate, although the numbers receiving prescriptions for non-alendronate bisphosphonates were small and only the association with alendronic acid reached statistical significance. The association between prior bisphosphonate use and incident acute pseudogout was seen in females but not males.

Previous evidence of acute pseudogout occurring as a complication of bisphosphonate treatment is limited to anecdotal case reports.^[9–14]^ Although our study design cannot establish causation, it confirms a clear temporal relationship between bisphosphonate prescription and subsequent incident acute pseudogout suggested by these earlier reports. Furthermore, plausible biological mechanisms exist by which bisphosphonates could predispose to CPPD.^[21–23]^ There were no exposed pseudogout cases who had initiated a bisphosphonate >60 days prior to the index date. This suggests that the acute effects of bisphosphonates on crystal shedding secondary to hypocalcemia^[21]^ might be more important than effects on pyrophosphate metabolism.^[22,23]^ A recent cross-sectional study reported an association between radiographic chondrocalcinosis and low cortical bone mineral density.^[33]^ This observation might suggest that the association between bisphosphonates and incident acute pseudogout could be explained by channeling bias and reflect a role for osteoporosis, the most common indication for bisphosphonate therapy, in the etiology of CPPD rather a specific drug-related effect. However, the authors suggested hyperparathyroidism as the most likely underlying mechanism, but in contrast to our study, were unable to adjust for this possible confounding relationship. Another possible approach to this conundrum would be to compare the risk associated with bisphosphonate use to other drugs used to treat osteoporosis (e.g., strontium ranelate). This was not feasible in our study since non-bisphosphonate osteoporosis treatments tend to be reserved for highly selected cases who have more severe disease or who fail to tolerate or have contra-indications to bisphosphonates, and are rarely prescribed in UK primary care.

We found a statistically significant association between incident acute pseudogout and prior alendronic acid prescription and also positive but nonsignificant associations for disodium etidronate, risedronate sodium, and sodium clodronate suggesting a possible class effect. Interestingly, pseudogout cases were less likely than controls to have been prescribed ibandronic acid, although the numbers receiving prescriptions were small and this association was not statistically significant. Published case reports describe acute pseudogout occurring following administration of oral alendronic acid and disodium etidronate, and intravenous pamidronate and neridronate.^[9–14]^ but there are no published reports of acute pseudogout following administration of ibandronic acid. We are unable to definitively explain this observation although adherence may be lower with monthly bisphosphonate preparations such as ibandronic acid.^[34]^ It is also possible that prescriptions for monthly drugs such as ibandronic acid could be issued for longer periods, for example, 3 or 6 months, and hence prescriptions issued before the 60-day exposure window would not have been captured. Patients receiving ibandronic acid were likely to have been previously prescribed alendronic acid (16 out of 24 patients prescribed ibandronic acid) raising the additional possibility that individual cases predisposed to CPPD had already experienced incident acute pseudogout following previous treatment with alendronic acid.

While our findings raise the possibility of bisphosphonates as a cause of pseudogout, this should not deter clinicians from prescribing them where the risk of fracture warrants their use. Indeed, a Cochrane review estimated that the number needed to treat with alendronic acid for the secondary prevention of vertebral fracture in postmenopausal women is 16,^[35]^ while using an estimated prevalence of pseudogout 1.6% in the unexposed, as in our sample size calculation, and our unadjusted IRR of 1.69, we estimate the number needed to harm to be 91.^[36]^

By nesting our study within the UK-CPRD and selecting all cases of incident pseudogout within the database during the study period, our findings should be generalizable to the wider UK primary care population. Matching cases and controls by age, sex, and GP practice reduces the likelihood of confounding by sociodemographic characteristics. A number of limitations of our study are worthy of acknowledgment. First, cases were identified by a pseudogout Read code rather than the gold standard of microscopic identification of CPP crystals in aspirated synovial fluid risking misclassification bias.^[1]^ However, our experience is that pseudogout is rarely diagnosed by general practitioners in the UK and it is likely that a pseudogout Read code would be most commonly entered following assessment in secondary care where synovial fluid aspiration and crystal identification is more commonly performed. It is possible that other conditions, particularly gout, could have been misdiagnosed as pseudogout. For this reason, we undertook a sensitivity analysis excluding participants with a previous diagnosis of gout, as previously performed in another UK primary care database study of risk factors for pseudogout,^[8]^ without changing our findings. Given that pseudogout is rarely diagnosed in primary care, it is perhaps more likely that pseudogout is misclassified as another diagnosis, such as gout, which would bias our findings toward the null hypothesis. Second, our exposure definition did not include intravenous bisphosphonates. We chose to limit our exposure to oral bisphosphonates because intravenous bisphosphonates are not prescribed in primary care in the UK and the majority of bisphosphonates are orally administered.^[18]^ Third, we were unable to assess bisphosphonate adherence which is often poor. One-third to one-half of patients are reported to not take bisphosphonates as directed.^[37]^ However, failure to identify cases of pseudogout misclassified as having gout, nonascertainment of prescription of intravenous bisphosphonates, and poor adherence would bias our findings toward the null hypothesis. Fourth, there remains a possibility of residual confounding. We adjusted for known risk factors for pseudogout but were unable to adjust for hypomagnesemia.^[3,4]^ However, we did adjust for use of diuretics which are thought to predispose to CPPD via enhanced renal magnesium loss.^[2,8]^ A 5th caveat is the possibility that delay in recording the clinical onset of acute pseudogout in the primary care medical record could mean that bisphosphonate initiation actually occurred after the onset of acute pseudogout. However, an additional sensitivity analysis restricting the definition of bisphosphonate exposure to those who had newly initiated a bisphosphonate in a stricter time period of 4 weeks prior to the index date did not significantly alter our findings (data not shown). Furthermore, such delay in recording the pseudogout diagnosis should not differ between those exposed and not exposed to a bisphosphonate. Finally, the absolute numbers of cases and controls in younger age-groups were small raising the possibility that the lack of significant interaction for age is a type II error, reflecting insufficient statistical power.

Further research is needed to confirm our findings using a robust case definition requiring identification of CPP crystals. The risks of CPPD associated with osteoporosis and use of intravenous bisphosphonates are worthy of exploration. The relative risks conferred by different individual bisphosphonates and the possible protective effect of ibandronic acid also warrant investigation in a large adequately powered study. Finally, the mechanisms by which bisphosphonates predispose to CPPD require further elucidation. The main clinical implication of our findings is that prescribers should be aware of acute pseudogout as a possible side effect of bisphosphonate treatment. People presenting with acute pseudogout should undergo medication review to look for bisphosphonates as a possible cause. Similarly, use of bisphosphonates should be considered carefully in people with known CPPD.
